# A Risk Prediction Index for Amiodarone-Induced Thyrotoxicosis in Adults with Congenital Heart Disease

**DOI:** 10.1155/2012/210529

**Published:** 2012-02-12

**Authors:** Marius N. Stan, Erik P. Hess, Rebecca S. Bahn, Carole A. Warnes, Naser M. Ammash, Michael D. Brennan, Prabin Thapa, Victor M. Montori

**Affiliations:** ^1^Department of Internal Medicine, Division of Endocrinology, Mayo Clinic, Rochester, MN 55905, USA; ^2^Department of Emergency Medicine, Mayo Clinic, Rochester, MN 55905, USA; ^3^Department of Internal Medicine, Division of Cardiovascular Diseases, Mayo Clinic, Rochester, MN 55905, USA; ^4^Department of Health Sciences Research, Division of Biostatistics, Mayo Clinic, Rochester, MN 55905, USA

## Abstract

Amiodarone therapy in adults with congenital heart disease (CHD) is associated with a significant risk of amiodarone-induced thyrotoxicosis (AIT). We developed a risk index to identify those patients being considered for amiodarone treatment who are at high risk for AIT. We reviewed the health records of adults with CHD and assessed the association between potential clinical predictors and AIT. Significant predictors were included in multivariate analyses. The parameter estimates from multivariate analysis were subsequently used to develop a risk index. 169 adults met eligibility criteria and 23 developed AIT. The final model included age, cyanotic heart disease and BMI. The risk index developed identified 3 categories of risk. Their AIT likelihood ratios were: 0.37 for low risk (95% CI 0.15–0.92); 1.12 for medium risk (95% CI 0.65–1.91); and 3.47 for high risk (95% CI 1.7–7.11). The AIT predicted risk in our population was 5% for the low risk group, 15% for the medium risk group and 47% for the high risk group. *Conclusions*. We derived the first model to quantify the risk for developing AIT among adults with CHD. Before using it clinically to help selecting among alternative antiarrhythmic options, it needs validation in an independent population.

## 1. Introduction

In the United States, the population of adults with congenital heart disease (CHD) is estimated at approximately 1 million, [1, 2], and the prevalence of those with complex CHD is growing at a rate of about 5% per year [1]. These individuals experience tachyarrhythmias, mainly atrial, at > 20% prevalence with more than double the risk of adverse events (stroke, heart failure) compared to those without arrhythmia [3]. The most commonly used therapies are radiofrequency ablation (RFA) and antiarrhythmic drugs. Unfortunately RFA is often made difficult by the complex cardiac anatomy of these patients, and antiarrhythmic medications carry significant risks associated with the specific medications used. Negative inotropic action, a common side effect of antiarrhythmic medications, is potentially harmful to patients with impaired ventricular function. For many of these patients with moderate and complex CHD, maintenance of sinus rhythm is essential [4].

Amiodarone is one of the agents with very good efficacy for arrhythmia control and is hemodynamically well tolerated by patients with impaired inotrope function and/or preexistent hypotension. Amiodarone, however, is associated with a number of side effects [5] including amiodarone-induced thyrotoxicosis (AIT). Given the morbidity and mortality associated with AIT [6], a clinical risk index that would inform clinicians and their patients regarding the probability of developing AIT could prove clinically useful, enabling risk-informed decision making. (See supplementary available online at doi:10.1155/2012/210529 for two cases scenarios that illustrate the potential utility of an AIT risk index). In this study we sought to develop an AIT risk index for use in CHD patients with tachyarrhythmias who are being considered for amiodarone therapy. 

## 2. Methods

### 2.1. Study Design and Setting

 We conducted a single-center health records review of consecutive patients presenting to the adult CHD clinic at Mayo Clinic Rochester. The Mayo Clinic Institutional Review Board approved the protocol without the need for informed consent. Records of patients who had not given prior authorization to have their medical records reviewed for research purposes were excluded in compliance with Minnesota Statute. The authors of this manuscript have certified that they comply with the Principles of Ethical Publishing [7].

### 2.2. Database Used

 From the database of Adult CHD Clinic at Mayo Clinic Rochester, we identified a cohort of CHD patients treated with amiodarone during the period 1987–2009. The Adult CHD Clinic is an academic tertiary referral practice, and the database contains demographic information, diagnoses, and medication lists for all patients with CHD seen in the Clinic. A team of 6 trained nurse abstractors, blinded as to patient outcome, abstracted additional data from patients' electronic and paper health records using a standardized data abstraction form. The nurse abstractors were trained by means of a 1-hour training session, and periodic meetings were held thereafter with the lead investigator (M. N. Stan) to address any questions that arose during the abstraction process. To validate the abstracting procedure, the lead abstractor (“gold standard”) reabstracted a random sample of 10 patients. We identified 24 differences in the 330 values reviewed indicating that the abstracting process had an acceptable accuracy of 92.7%.

### 2.3. Patient Selection and Timing of Follow-Up Assessment

 From the complete database (4,883 patients), we selected for further study patients who received amiodarone for a minimum of 90 days and had no prior history of hyperthyroidism or thyroidectomy. We followed the patients until their final thyroid evaluation on amiodarone or until they developed AIT.

### 2.4. Outcome Measures and Definitions

AIT was defined as suppressed TSH with an elevated or normal T4 and/or T3 in the outpatient setting or suppressed TSH with elevated T4 and/or T3 in the inpatient setting with the exclusion of sick euthyroid and/or drug effects (i.e., heparin, dopamine, and glucocorticoids). Investigators assessed for this outcome unaware of which features would be selected for predictor variables. The two criteria to be met for AIT type 2 were [8, 9] (a) absence of nodular goiter or presence of small diffuse goiter with negative thyroid antibodies and (b) RAI uptake <3% and low vascularity on thyroid Doppler flow (if only one investigation was available, it was accepted as sufficient for this criteria). If one of these criteria was not met, the AIT was defined as type 1. If there was not sufficient information to make this determination, the AIT was labeled as “undefined.” Cyanotic CHD was defined as a congenital heart anomaly with a right-to-left shunt that either did not undergo surgical repair or had an incomplete or palliative repair prior to amiodarone therapy.

### 2.5. Statistical Analysis

Summary statistics for the continuous variables were expressed as mean and standard deviation (SD) or median with interquartile range (IQR), depending on the normalcy of distribution. Categorical variables were summarized as percentages. Comparison between groups was based on a two-sample *t*-test for continuous variables and Pearson's chi square test for categorical variables. Bivariate and multivariate predictors of AIT development were assessed with the Cox proportional hazard model using SAS*©* software version 9.2. Results of the analyses were summarized as relative risk (RR), hazard ratio (HR), and likelihood ratio (LR) with their 95% confidence intervals (CI) and *P*-values. To address the skewed distribution for BMI, ordinal levels were computed for the multivariate analysis, assigning lowest level for the high BMI subgroup. *P* values were reported with 3 digits if <0.1.

## 3. Results

We identified 23 AIT cases from the cohort of 169 CHD patients. The median follow-up period was 3.1 years (IQR 1.5–6.7 years). There were 2 patients for whom BMI and dose of amiodarone were not available, leaving 167 patients for inclusion in the risk factor analysis.

In these analyses the 2 clinical AIT subgroups (type 1 with 7 patients and type 2 with 13 patients) had similar BMI at initiation of amiodarone (type 1: mean BMI 22.2, SD 3.4; type 2: mean BMI 21.5, SD 3.1; *P* = 0.71) as well as with similar gender, age, cyanotic CHD prevalence, average amiodarone dose, and duration of therapy (*P* ≥ 0.1 for each). Three cases had an undefined AIT subtype. In a sensitivity analysis, we assigned them to the most likely category based on the available data; this did not change the BMI comparison between the AIT subtypes. Due to these similarities, all AIT subtypes were combined for further risk factor analysis. Twenty-one variables were assessed for association with AIT (Table 1).

The variables most likely to be predictive of AIT were assessed in a bivariate Cox proportional hazard model (Table 2).

After these bivariate analysis, a number of parameters were included in the multivariate analysis based on their results in the initial analyses, their biological association with the outcome, and on associations reported in the literature (see supplementary Table  1). These analyses were carried out using the Cox proportional hazard method. For this analysis we selected BMI in the 3 categories (high if >25, normal for 21 to 25, and low if <21), gender, cyanotic status, age at start of amiodarone therapy, goiter, and average amiodarone dose mg/kg body weight. Duration of amiodarone therapy was implicit in the Cox model. In multivariate analyses we observed BMI in 3 categories (HR estimate 3.2 with 95% CI = 1.6–6.2) and goiter (HR estimate 5.6 with 95% CI = 2–16) to be the only independent predictors of AIT. As goiter was associated only with AIT type 1 in this cohort and its presence usually excludes AIT type 2 [10], it was excluded from consideration for the final model. Age was included in the final model due to its confounding effect on BMI (Pearson's correlation *r* = 0.24 with *P* = 0.007). Cyanosis showed a trend for association with BMI in our bivariate analysis (*P* = 0.059) and was reported in a similar cohort to be predictive of AIT [11]. These consistencies along with its biological plausibility [12] led us to include cyanosis in the final model. The final analysis is displayed in Table 3.

### 3.1. Baseline Risk Calculator

From the final model we developed an overall AIT risk score at initiation of amiodarone. This calculation was based on parameter estimates, from the multivariate Cox proportional hazard model. Based on the ratio between the parameter estimates, the following method was used to allocate points for the risk score: 2 points were allocated for each BMI category increase over category “0” (i.e., BMI > 25) and 1 point was allocated for patients with cyanotic CHD (category “1”) in contrast to 0 points for the noncyanotic patients (category “0”). The range of possible scores is 0 to 5. The distribution of patients according to their risk score is presented in supplementary e-Table  2. The higher the score, the higher the likelihood for that patient to develop AIT in the future. The model generated the following formula for risk calculation:

(1) $$\begin{array}{cc} \text{AIT}\;\text{Risk}\;\text{prediction}\;\text{index} & =\text{BMI}\;\text{category}\;\times \;2\\ & \;+\;\text{Cyanotic}\;\text{category}\;\times \;1. \end{array}$$

The likelihood ratios (LRs) for the different levels of risk are included in Table 4.

 In order to improve the precision of our risk estimate and given the close results observed between consecutive scores, we combined scores 0 and 1 in the low-risk category, scores 2 and 3 in the medium-risk category, and scores 4 and 5 in the high-risk category. Using the known cumulative incidence of AIT in a CHD population, one can calculate the actual risk of AIT for a given individual. Starting with a pretest probability of AIT development of 13.6% (the cumulative incidence in our population), we calculated a posttest probability of AIT of 5.0% for the low-risk group, 15.2% for the medium-risk group, and 47.2% for the high-risk group.

**Figure 1:**
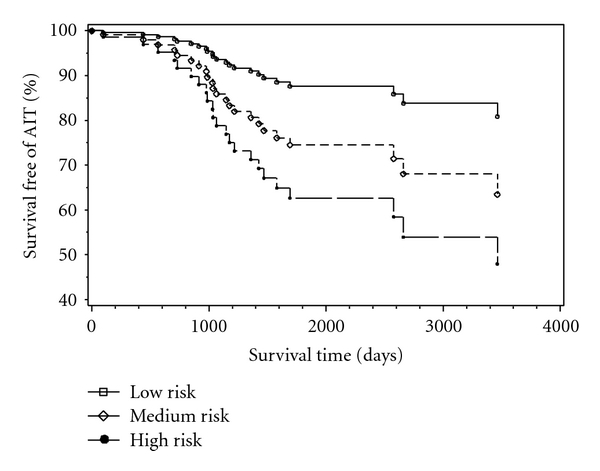
Percent survival free of AIT (%) based on length of time on amiodarone and AIT risk category.

Since AIT development is time dependent, we also calculated the actual risk for each category in relationship to the expected time the patient will be on amiodarone (Figure 1 and see supplemetary e-Table  3).

## 4. Discussion

We developed a risk prediction index that can determine the likelihood of AIT in adults with CHD. We identified a cohort of 169 adults with CHD who were treated with amiodarone for at least 90 days, among which 23 (13.6%) developed AIT. Among the 5 variables significantly associated with AIT on univariate analysis, we developed a multivariate model that includes age, cyanosis, and BMI at initiation of amiodarone to predict an individual patient's risk of developing AIT. These variables are simple to collect and readily available in the outpatient setting. To the best of our knowledge, this is the first study in which a risk prediction index for AIT in adults with CHD was developed. Our prediction model requires validation in an independent patient population before implementation in clinical practice. The potential utility of the risk calculator is demonstrated in two case studies attached in the online Appendix.

 A number of individual risk factors for AIT have been identified in the general population including low-iodine status [13], male gender [14, 15], and goiter, which was found to be present in 67% of AIT cases in one series [16]. A specific risk factor for patients with CHD is cyanotic CHD status [11]. In our cohort we also identified low BMI as a risk factor for AIT and noted the confounding effect that age has on this variable.

The explanation for this novel association likely relates to the preferential deposition of amiodarone in adipose tissue combined with its concentration-dependent cytotoxicity [17, 18] and the current clinical practice of giving a fixed, rather than a weight-adjusted, dose of amiodarone. We suggest that the lipophilic character of amiodarone leads to lower drug exposure (i.e., lower blood and tissue levels) into the thyroid in obese individuals and to higher drug exposure (i.e., higher blood and tissue levels) in individuals with smaller adipose tissue depots, making the latter at higher risk for AIT. Prospective studies to determine the impact of BMI on serum and tissue amiodarone levels at steady state are warranted to further explore this hypothesis.

To date there are no published data that assemble these various risk factors into a comprehensive risk assessment index that could be used to gauge an individual's risk for AIT. The current report represents the first risk prediction index based on variables associated with AIT development in this population. It is notable that this calculator adds significant information to the risk assessment by adjusting the average population risk to the individual patient. In doing so, a patient's calculated risk may range anywhere between 5.0% and 47.2% at the extremes of the risk index. If validated, the results we report as LRs could be used in similar populations provided that the prevalence of disease can be estimated. If so LRs can serve to calculate the posttest probability of AIT for that population [19]. It is important to refine the AIT risk because of the high morbidity and mortality associated with AIT [6]. This risk index could become a very useful tool in quantifying the individual benefits and risks associated with amiodarone use and thus supporting clinical decision making. Identifying a high risk of AIT could lead to the decision to use a different antiarrhythmic agent without thyroid side effects [20], to attempt cardioversion or to attempt ablation of the arrhythmogenic focus [21].

In order to construct the instrument, we opted for Cox proportional hazard analysis as opposed to recursive partitioning given our aim for high overall accuracy where both low and high risk for AIT are important outcomes in our cohort [22]. In order to increase the reproducibility of our findings, we used a very clear definition for the outcome based on biochemical data. All the parameters that were selected for the model [23] are either biologically connected with the outcome (time on amiodarone and age of patient) and/or have demonstrated association with AIT in our cohort analysis (BMI and cyanotic status). Some parameters known to be associated with AIT, including goiter and low-iodine status, were not included in this instrument. Goiter is highly predictive of type 1 AIT and, by definition, excludes the identification of most AIT type 2 cases. Since the latter is the dominant form of AIT [10], we have decided against including goiter in the final Cox model since it would bias the model to calculate a lower risk for those likely to develop AIT type 2. Low-iodine status as a risk factor for AIT has been reported in earlier studies [13]. While iodine status was not specifically assessed in our population, our cohort was composed almost exclusively of US residents who are likely to be iodine sufficient [24]. Therefore, our results may not generalize to an iodine-deficient population.

We believe that our instrument is easy to use given the limited number of clearly defined, clinically relevant variables that must be collected. It is also, clinically sensible, and likely to be incorporated easily into the flow of patient care. However, it must first be validated in an independent patient population before it can be used in the clinical setting.

Our study has a number of limitations. The study design was a health records review and is susceptible to the types of biases common to this study design. However, we took several steps to limit the intrusion of bias into the data abstraction process including training of data abstractors, using a standardized data abstraction form, uniform definition of important predictor variables, clearly defined outcomes, assessment of the accuracy of data abstraction, blinding abstractors as to outcome at the time of predictor variable assessment and to study hypothesis, blinding investigators to the predictor variables at the time of the outcome assessment, and periodic meetings to address questions that arose in the process of data abstraction. Given the time required to develop AIT, a health records review design is the most pragmatic and economically efficient design. We ascertained the predictor variables age, BMI, and cyanotic status of the CHD in 167/169 patients from our cohort (98.8%), minimizing thus the risk of selection bias. There were also only 23 patients in the cohort who developed AIT, increasing the risk of overfitting our model. Validation of our findings in another cohort of adults with CHD will allow for refinement and validation of the model. Another limitation was that we could capture only presence or absence of goiter, rather than more accurate information concerning thyroid volume as only a minority of patients in our cohort underwent thyroid ultrasound. For similar reasons, we were unable to carry out an analysis addressing the impact of serum amiodarone levels on the AIT risk, as this parameter was obtained in only approximately 10% of the cohort. Future prospective studies will better quantify these elements and allow full exploration of mechanisms underlying our findings.

In conclusion our results provide for the first time a quantification of the risk of AIT in amiodarone-treated adults with CHD. The identification of patients at high risk for AIT could lead the clinician to reconsider the antiarrhythmic choice and select a different agent or offer ablation of the arrhythmogenic focus if anatomically feasible. For patients unlikely to respond to other antiarrhythmic therapies, prophylactic thyroid ablation with radioactive iodine or thyroidectomy might be considered. Another potential consideration, owing to the relatively high risk associated with low BMI, might be to weight-adjust the dose of amiodarone in patients with low BMI in order to minimize the tissue cytotoxicity of the drug. We acknowledge that our risk prediction index is not yet ready to be used clinically; it has been derived only and is level 4 evidence as classified by McGinn et al. [25]. Validation in an independent patient population is required before the index can be used in clinical practice.

## Supplementary Material

The following section describes 2 clinical cases of patients with CHD that are being evaluated with consideration for starting amiodarone therapy. Based on the clinical data the AIT risk index is calculated and then the implication of that result is discussed.

## Figures and Tables

**Table 1:** Overall cohort and group characteristics (*n* = 169).

Variable	Entire cohort (*n* = 169)	Group with AIT	Group without AIT	*P* value
Men—*n* (%)	89 (52.7)	13 (56.5)	76 (52.1)	0.69
Age (years)—mean (SD)	42.7 (14.1)	35.3 (12.2)	43.8 (14.1)	0.006
BMI (kg/m^2^)—mean (SD)	25.2 (4.9)	22.0 (3.1)	25.8 (4.9)	<0.001
Weight (kg)—mean (SD)	69.9 (16.5)	62.2 (11.8)	75.5 (16.5)	0.001
Supraventricular arrhythmia*—*n* (%)	152 (90)	23 (100)	129 (88.4)	0.084
Baseline systolic blood pressure (mm Hg)—mean (SD)	117 (13)	116 (17.5)	117.2 (12.5)	0.77
Cyanotic CHD diagnosis—*n* (%)	34 (20.1)	8/23 (34.8)	26/146 (17.8)	0.059
Tricuspid regurgitation—*n*(%)				
Absent Mild Moderate Severe	37 (25) 50 (35) 23 (16) 34 (24)	7 (37) 7 (37) 2 (10.5) 3 (15.8)	30 (24) 43 (34) 21 (16.8) 31 (24.8)	0.17**
Left ventricular ejection fraction % —mean (SD)	47.5 (13.5)	43 (13.2)	48 (13.4)	0.15
Hematocrit (%)—mean (SD)	42.9 (7.2)	43.5 (4.7)	42.8 (7.5)	0.61
Protein-losing enteropathy—*n* (%)	8 (4.8)	0 (0)	8 (5.5)	0.6
Goiter—*n* (%)	19 (12.9)	7 (35)	12 (9.5)	0.002
Albumin (g/dL)—median (IQR)	4.3 (4.0–4.5)	4.4 (4.2–4.7)	4.3 (4–4.5)	0.13
Average amiodarone dose/day (mg/day)—median (IQR)	200 (200–300)	200 (200–250)	200 (200–300)	0.77
Average amiodarone dose/kg/day (mg/kg/day)—median (IQR)	3.04 (2.42–3.87)	3.51 (2.32–3.85)	2.96 (3.05–4.12)	0.015
Time on amiodarone (days)—median (IQR)	1147 (546–2438)	1066 (849–1470)	1206 (393–2529)	0.79
Cumulative amiodarone exposure (g/kg)—median (IQR)	3.7 (1.2–7.3)	3.5 (2.7–5.2)	3.8 (1.1–7.3)	0.60
Abnormal liver tests—*n* (%)	26 (16.5)	2 (8.7)	24 (17.8)	0.27
Abnormal pulmonary function test—*n* (%)	67 (84.4)***	14 (100)	53 (81.5)	0.11
Hospital admissions per year on amiodarone****—median (IQR)	0.4 (0.2–1.1)	0.4 (0.1–1.0)	0.6 (0.2–1.1)	0.57
Days in the hospital per year on amiodarone****—median (IQR)	3.4 (1.2–8.0)	2.6 (0.9–6.4)	4.5 (1.7–13.5)	0.15

* The ventricular arrhythmias treated were ventricular tachycardia, premature ventricular contractions, and ventricular fibrillation.

**P value for trend.

*** This variable was available in less than 75% of the entire cohort.

**** 23 age and gender matched pairs used for this analysis.

BMI: body mass index; CHD: congenital heart disease.

**Table 2:** Bivariate models for AIT risk prediction (Cox proportional hazard model).

Risk factors	Parameter estimate	*P* value (chi square)	HR	95% CI of HR (LL, UL)	
Age at start of amiodarone	−0.026	0.145	0.974	0.941	1.009
Amiodarone dose mg/kg	0.241	0.167	1.272	0.904	1.789
BMI (3 categories)	0.966	0.002	2.627	1.438	4.798
BMI (>25)	−1.903	0.002	0.149	0.045	0.499
BMI (21 to 25) (reference = BMI < 21)	−1.047	0.034	0.351	0.134	0.921
Cyanotic	0.630	0.135	1.878	0.822	4.293
Goiter	1.405	0.003	4.08	1.6	10.3
Gender (reference = female)	0.019	0.964	1.02	0.45	2.3

BMI: body mass index; CI: confidence interval; HR: hazard ratio; LL: lower limit; UL: upper limit.

**Table 3:** Final Cox proportional hazards model for development of AIT.

Risk factor of AIT	Parameter estimate	*P*-value (chi square)	HR		
			Estimate	95% LL	95% UL		
Age	−0.02	0.266	0.979	0.942	1.017		
Cyanotic (ref: cyanosis = no)	0.43	0.345	1.536	0.631	3.74		
BMI 3 categories (ref: BMI > 25)	0.83	0.009	2.286	1.226	4.262		

**Table 4:** Likelihood ratios according to AIT risk score.

Risk score	*P* (disease)	*P* (no disease)	Likelihood ratio (95% CI)	Combined categories	Likelihood ratio (95% CI)
0	3/21 = 14.3%	60/146 = 41.4%	0.34 (0.12–1.01)	Low risk	0.37 (0.15–0.92)
1	1/21 = 4.8%	14/146 = 9.6	0.49 (0.07–3.58)		
2	5/21 = 23.8%	42/146 = 28.8	0.83 (0.37–1.85)	Medium risk	1.12 (0.65–1.91)
3	4/21 = 19.0%	14/146 = 9.59	1.98 (0.72–5.47)		
4	3/21 = 14.3%	8/146 = 5.5%	2.61 (0.75–9.06)	High risk	3.47 (1.70–7.11)
5	5/21 = 23.8%	8/146 = 5.5%	4.35 (1.57–12.04)		

## Abbreviations

AIT: Amiodarone-induced thyrotoxicosis
BMI: Body mass index
CHD: Congenital heart disease
LVEF: Left ventricular ejection fraction
TSH: Thyroid-stimulating hormone
T4: Thyroxine
T3: Triiodothyronine
LR: Likelihood ratio.
