# Supplementary material for: A Risk Prediction Index for Amiodarone-Induced Thyrotoxicosis in Adults with Congenital Heart Disease
Source: J Thyroid Res. 2012 Feb 12;2012:210529. doi: 10.1155/2012/210529 (PMC3306911; doi:10.1155/2012/210529)
Supplement: Supplementary file 1 — The following section describes 2 clinical cases of patients with CHD that are being evaluated with consideration for starting amiodarone therapy. Based on the clinical data the AIT risk index is calculated and then the implication of that result is discussed. [file 210529.f1.pdf]

Cases of adults with CHD considering amiodarone therapy and the use of risk prediction index

Case 1 - A 34 year-old man with transposition of the great arteries, s/p Rashkind atrial septostomy x 2 and Mustard procedure, is seen in follow-up at the adult Congenital Heart Disease (CHD) Clinic. He has a history of tachyarrhythmias, predominantly supraventricular, for which he was tried successively on propranolol, digoxin, quinidine and sotalol. He also has significant pulmonary hypertension with a pulmonary artery pressure of 81/38 mm Hg with severe elevation of pulmonary vascular resistance. His tachyarrhythmias have been relatively well controlled on sotalol until last month when he had 3 episodes of tachyarrhythmias necessitating hospitalization. Adenosine injection, vagal maneuvers and metoprolol were ineffective and he was loaded with amiodarone for 10 days. Because of the side-effects of amiodarone he was counseled on the possible need for radiofrequency ablation and seeks an opinion regarding further management of his arrhythmias. Due to his residual atrial septal defect he still has cyanotic CHD which is noted on exam with finger oximetry at 92% on 2 liter/minute per nasal cannula. He is a lean individual with a BMI of 19.6 and no evidence of goiter on examination. Liver is 1-2 cm below costal margin and his jugular venous pressure is about 8 cm. Cardiac examination documents a healed sternotomy, active precordium, normal S1, P2 slightly accentuated, a very soft systolic murmur at the left lower sternal border and no diastolic murmur, rub, click or gallop. The rest of the exam is unremarkable.

Case 2 - A 31-year-old man with history of transposition of the great vessels with multiple surgical interventions (Rastelli procedure complicated by homograft stenosis, replacement of right ventricle to pulmonary artery conduit with Hancock conduit, Hancock conduit stenosis with conduit excision and pericardial patch reconstruction and DeVega tricuspid annuloplasty) has a long history of atrial fibrillation. The atrial fibrillation has been managed with verapamil, diltiazem and most recently with sotalol. He developed dyspnea on exertion and increased the number of pillows he sleeps on from 2 to 3. Due to his general decline he presented for reevaluation. He is overweight (BMI 29.5), with +1 peripheral edema but in no acute distress. His jugular venous pressure is 18 cm. There is prominent right ventricular lift with soft S1 and S2. There is a pan systolic grade 2/6 murmur at the left sternal border which increases with inspiration and grade 3/6 decrescendo diastolic murmur in the 2nd left intercostal space. The electrocardiogram confirms that he is in atrial fibrillation, while the echocardiogram reveals marked cardiomegaly with biventricular enlargement and decreased systolic function for both ventricles. At the conclusion of the evaluation the consulting physician decided that owing to his congestive heart failure, the indicated medical therapeutic options for his atrial fibrillation were quinidine or amiodarone.

For both of these patients, the possible development of AIT with subsequent cardiovascular deterioration would represent a particularly serious a side-effect of amiodarone therapy. While it is known that the reported prevalence of AIT in the CHD population is approximately 20 % (1), one wonders whether the personal risk for AIT in

each of these patients could be better predicted in order to aid in decision making regarding long-term management of their tachyarrhythmias.

The risk index calculator that we created is:

AIT Risk score = BMI category × 2 + Cyanotic category × 1

Let’s see how that would work in the 2 cases that we presented earlier:

Case 1: The patient was treated with amiodarone for 2.8 years at a dose of 200 mg / day. He developed pronounced dyspnea which was presumed to be related to amiodarone-induced pulmonary dysfunction and persistent tachyarrhythmias, and amiodarone was discontinued. He was found to be severely thyrotoxic and was diagnosed as having AIT. He had significant resistance to medical therapy for hyperthyroidism and required radiofrequency catheter ablation followed by AV node ablation and trans-venous dual chamber pacing implantation for tachyarrhythmia control. His hyperthyroidism resolved slowly over 6 months and his overall health remained very precarious. Based on our risk assessment his AIT risk index at initiation of amiodarone was 5, equivalent of a high risk index. With a likelihood ratio (LR) of 3.47 and a prevalence of disease in this population at 13.6% his post-test probability of developing AIT was 47.2%. This very high risk might have led to the selection of a different anti-arrhythmic therapy.

Case 2: The patient was started on amiodarone and treated with a maintenance dose of 200 mg per day. He has been followed so far for 8.3 years on amiodarone and developed no thyroid dysfunction. Based on our risk assessment his AIT risk index was 0, equivalent to a low risk index. With the LR of 0.37 and the prevalence of disease in this

population of 13.6, his post-test probability of developing AIT was 5.0 %. This low-risk would likely reassure the patient and the physician that amiodarone therapy is unlikely to create significant endocrine problems.

These 2 cases exemplify the extreme differences in AIT risk that can be detected by our risk prediction calculator between individuals with CHD. Once the instrument is validated these differences can be used in clinical practice for decision making regarding amiodarone therapy in comparison with alternative anti-arrhythmic therapies.

E-Table 1 - Multivariate models for AIT risk prediction (Cox proportional hazard model)

Number of variables	Model number	Risk factors	Parameter estimates	P value (chi-square)	HR	95% CI of HR (LL, UL)	
2 variable models	Model 1	Age at amiodarone start	-0.03	0.17	0.97	0.94	1.01
		amiodarone dose mg/kg	0.18	0.32	1.19	0.84	1.69
	Model 2	Age at amiodarone start	-0.02	0.27	0.98	0.94	1.02
		BMI (3 categories)	0.91	0.004	2.48	1.34	4.58
	Model 3	Age at amiodarone start	-0.02	0.17	0.98	0.94	1.01
		Cyanotic	0.65	0.13	1.91	0.82	4.44
	Model 4	amiodarone dose mg/kg	0.07	0.75	1.07	0.71	1.61
		BMI (3 categories)	0.92	0.005	2.52	1.33	4.77
	Model 5	amiodarone dose mg/kg	0.17	0.32	1.19	0.85	1.68
		Cyanotic	0.67	0.140	1.95	0.80	4.74
	Model 6	BMI (3 categories)	0.88	0.005	2.41	1.30	4.48
		Cyanotic	0.47	0.31	1.59	0.65	3.89
3 variable models	Model 1	Age at amiodarone start	-0.02	0.27	0.98	0.94	1.02
		amiodarone dose mg/kg	0.03	0.88	1.03	0.69	1.55
		BMI (3 categories)	0.89	0.007	2.42	1.28	4.61

	Model 2	Age at amiodarone start	-0.03	0.18	0.98	0.94	1.01
		amiodarone dose mg/kg	0.12	0.51	1.12	0.80	1.59
		Cyanotic	0.65	0.15	1.91	0.79	4.63
	Model 3	Age at amiodarone start	-0.02	0.28	0.98	0.94	1.02
		BMI (3 categories)	0.83	0.01	2.29	1.22	4.28
		Cyanotic	0.45	0.32	1.58	0.65	3.84
	Model 4	amiodarone dose mg/kg	0.04	0.85	1.04	0.70	1.56
		BMI (3 categories)	0.86	0.009	2.37	1.24	4.52
		Cyanotic	0.43	0.34	1.54	0.63	3.80
4 variable model	Model 1	Age at amiodarone start	-0.02	0.27	0.98	0.94	1.02
		amiodarone dose mg/kg	0.01	0.96	1.01	0.68	1.51
		bmi_cat3	0.82	0.013	2.27	1.19	4.35
		Cyanotic	0.43	0.35	1.53	0.63	3.75
	Model 2	Age at amiodarone start	0.007	0.72	1.0	1.0	1.0
		Cyanotic	0.57	0.25	1.8	0.7	4.6
		BMI (3 categories)	1.15	0.0009	3.2	1.6	6.2
		Goiter	1.73	0.0012	5.6	2.0	16.0

E-Table 2 - Patient distribution in the different AIT risk strata

**Risk strata**	**Cyanotic***	**BMI 3 categories****	**AIT (N)**	
**(0--5)**	**(0,1)**	**(0,1,2)**	**No**	**Yes**
0	0	0	60	3
1	1	0	14	1
2	0	1	42	5
3	1	1	14	4
4	0	2	8	3
5	1	2	8	5
		**TOTAL**	**146**	**21**

* Cyanotic: 0=No, 1=Yes

** BMI: 0= BMI>25; 1= BMI of 21-25; 2= BMI <21

E-Table 3 - Survival free of AIT (%) factoring time on amiodarone and AIT risk category

**Amiodarone duration (in Years)**	**AIT risk score = 0**	**AIT risk score = 1**	**AIT risk score = 2**	**AIT risk score = 3**	**AIT risk score = 4**	**AIT risk score = 5**
0.3	100	100	100	100	99	98
1.2	100	100	99	98	97	96
2	99	98	97	96	91	89
4.6	93	91	83	78	60	51
9.5	89	86	74	67	45	35

REFERENCES

1. Thorne SA, Barnes I, Cullinan P, Somerville J. Amiodarone-associated thyroid dysfunction: risk factors in adults with congenital heart disease. Circulation. 1999 Jul 13;100(2):149-54.
